# SAHA/5-AZA Enhances Acetylation and Degradation of mutp53, Upregulates p21 and Downregulates c-Myc and BRCA-1 in Pancreatic Cancer Cells

**DOI:** 10.3390/ijms25137020

**Published:** 2024-06-27

**Authors:** Michele Di Crosta, Francesca Chiara Ragone, Rossella Benedetti, Gabriella D’Orazi, Maria Saveria Gilardini Montani, Mara Cirone

**Affiliations:** 1 Department of Experimental Medicine, “Sapienza” University of Rome, 00161 Rome, Italy; michele.dicrosta@uniroma1.it (M.D.C.); ragone.1872024@studenti.uniroma1.it (F.C.R.); rossella.benedetti@uniroma1.it (R.B.); 2 Department of Neurosciences, Imaging and Clinical Sciences, University “G. D’Annunzio” Chieti, 66100 Pescara, Italy; gdorazi@unich.it; 3 Department of Research and Technological Innovation, IRCCS Regina Elena National Cancer Institute, 00144 Rome, Italy

**Keywords:** pancreatic cancer, acetylation, methylation, mutp53, c-Myc, DNA damage

## Abstract

Epigenetic changes are common in cancer and include aberrant DNA methylation and histone modifications, including both acetylation or methylation. DNA methylation in the promoter regions and histone deacetylation are usually accompanied by gene silencing, and may lead to the suppression of tumor suppressors in cancer cells. An interaction between epigenetic pathways has been reported that could be exploited to more efficiently target aggressive cancer cells, particularly those against which current treatments usually fail, such as pancreatic cancer. In this study, we explored the possibility to combine the DNA demethylating agent 5-AZA with HDAC inhibitor SAHA to treat pancreatic cancer cell lines, focusing on the acetylation of mutp53 and the consequences on its stability, as well as on the interaction of this protein with c-myc and BRCA-1, key molecules in cancer survival. The results obtained suggest that SAHA/5-AZA combination was more effective than single treatments to promote the degradation of mutp53, to upregulate p21 and downregulate c-Myc and BRCA-1, thus increasing DNA damage and cytotoxicity in pancreatic cancer cells.

## 1. Introduction

Histone deacetylase inhibitors (HDACi), which target with different specificities, class I (1, 2, 3 and 8), IIa (4 and 7), IIb (6 and 10), III (Sirtuins), and IV (11) histone deacetylases (HDACs) are emerging as promising anticancer drugs [1]. Among these, suberoylanilide hydroxamic acid (SAHA), also named vorinostat, inhibits classes I and II of HDACs. It was the first to be approved by the FDA for the treatment of advanced cutaneous T-cell lymphoma (CTCL) [2]. With the exception of class III, the deacetylating activity of all HDACi, including SAHA, is zinc-dependent, and in fact all these molecules contain a zinc-binding group (ZBG) [3]. In addition to histone acetylation, which determines chromatin relaxation and facilitates gene transcription, HDACi treatment can increase the acetylation of non-histone proteins. SAHA has also been reported to enhance the acetylating activity of drugs such as resveratrol, leading to acetylation of p53 and activating its pro-apoptotic function in prostate cancer cells [4]. Depending on treatments, p53 can be acetylated at multiple lysine sites, e.g., lys 381 and 382 by HDAC6 inhibitors [5], lys373 and lys382 by acetyltransferases such as p300 and CBP and lys320 by PCAF (p300/CBP associated factor) [6]. Together with phosphorylation at specific residues, acetylation leads to the activation of wtp53, even following treatments by DNA-damaging agents [7]. Moreover, HDAC6 inhibition has been reported to reduce the stability of mutant p53 (mutp53), by interfering with HSP90, leading to its proteasomal degradation [8]. Tumors, and pancreatic cancer, in particular, are characterized by numerous genetic abnormalities, including the p53 mutations, detected in more than 75% of pancreatic cancer cases [9]. Since p53 lysine acetylation does not occur in the DNA binding domain (DBD) where point mutations usually occur, this post-translational modification (PTM) does not distinguish between wt- and mutp53 and may therefore affect both proteins. It was recently shown that although both wt- and mutp53 can undergo acetylation, this PTM regulates their function in opposite ways, resulting in stabilization of wtp53 and destabilization of (R175H) mutp53, as demonstrated in non-small-cell lung cancer (NSCLC) [10]. This is particularly important, since p53, the most important gatekeeper of the genome, often undergoes missense mutations in tumor cells, resulting in the loss of DNA-binding ability and often in the acquisition of oncogenic properties (GOF) [11,12]. However, it remains to be investigated whether other mutant p53 proteins may be affected by acetylation in a similar manner and whether this may occur in tumor cells other than NSCLC. Besides acetylation, HDACi can influence DNA methylation, as they can for example inhibit DNA methyltransferase-3B, [13], resulting in a further activation of gene transcription. On the other hand, histone deacetylation can be influenced by DNA methylation, since DNA methyltransferase1 (Dnmt1), an enzyme that mediates DNA methylation, can also have histone deacetylation properties [14]. Therefore, DNA demethylating agents such as 5-AZA could indirectly interfere with HDAC activity. DNA methylation at promoter regions and histone deacetylation can both silence gene expression, thus dual inhibition of DNA methylation and histone acetylation has been reported to synergistically activate the metallothionein promoter in lymphosarcoma cells [15]. These proteins bind zinc, functioning as an intracellular zinc reservoir, and thus their upregulation can reduce the enzymatic activity of zinc-dependent HDACs, such as class II and class II HDACs [15]. Exploring the molecular mechanisms regulating the interconnection between DNA demethylation and HDAC inhibition is important, since the combination of drugs targeting both acetylation and methylation can synergistically reduce cancer survival, as for example they may reactivate oncosuppressors such as the phosphatase and tensin homologue (PTEN) [16]. In the present study, we investigated the impact of combining 5-AZA and HDAC inhibition by SAHA against pancreatic cancer cell lines, and focused on the effect on mutp53 acetylation and stability as well as on other molecules interconnected with mutp53 and strongly involved in carcinogenesis such as c-myc and BRCA-1.

## 2. Results

### 2.1. 5-AZA Enhances Pan-Lysine Acetylation and Cytotoxicity Induced by SAHA against Pancreatic Cancer Cells

**Figure 1:**
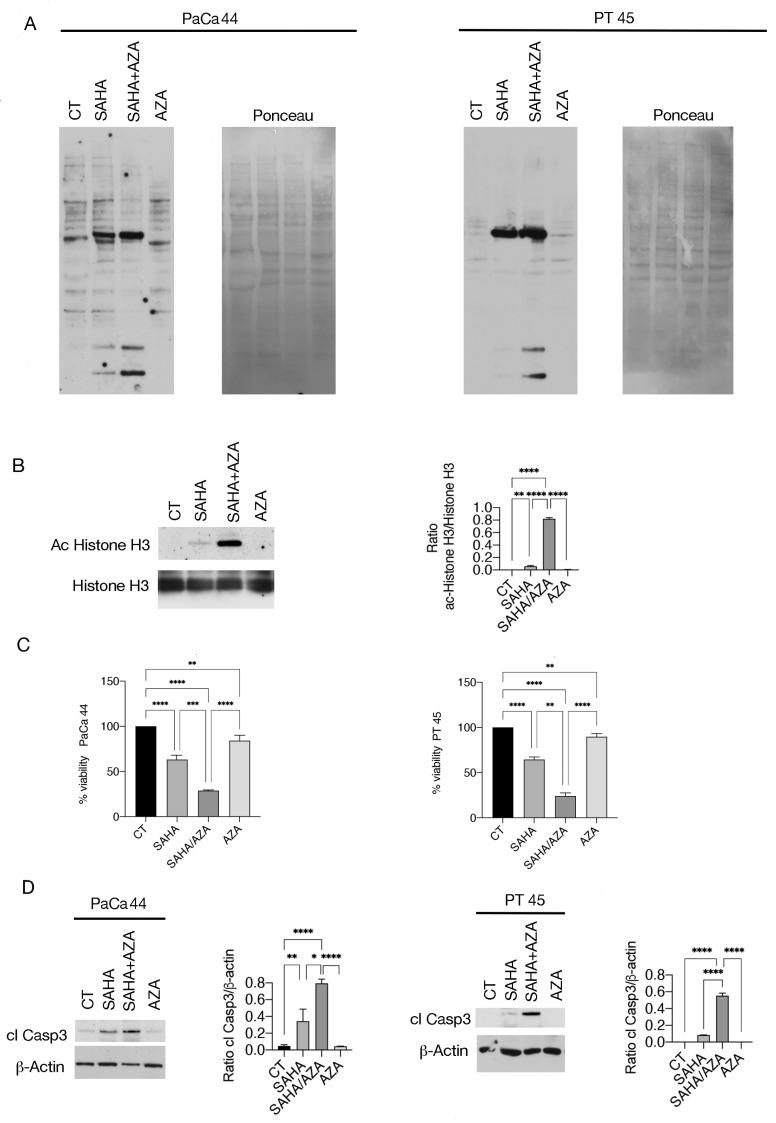
5-AZA increases acetylation and cytotoxicity more than SAHA in pancreatic cancer cells. (**A**) Acetylated proteins as evaluated by western blot analysis in PaCa44 and PT45 pancreatic cancer cell lines treated with SAHA (5 μM), 5-AZA (AZA) (40 nM), SAHA/AZA combination for 48 h or untreated (CT) by using an antibody against acetylated lysine (see material and methods). Ponceau staining is shown as loading control. (**B**) Acetylated Histone H3 expression level as evaluated by western blot analysis in PaCa44 cells treated as above reported. Histograms represent the mean plus SD of the densitometric analysis of the ratio of Acetylate Histone H3/Histone H3; *p*-value: ** < 0.01 and **** < 0.0001, as calculated by ANOVA test. (**C**) Cell survival was assessed by Trypan blue assay in PaCa44 and PT45 pancreatic cancer cell lines treated with SAHA, 5-AZA (AZA), SAHA/AZA combination at the same concentrations reported above, or untreated (CT). The histograms represent the percentage of cell viability relative to the control; data are shown as the mean plus SD of more than three experiments *p*-value: ** < 0.01; *** < 0.001; and **** < 0.0001, as calculated by ANOVA test. (**D**) Cleaved Caspase 3 (cl Casp3) expression level as evaluated by western blot analysis in PaCa44 and PT45 cells treated as above with SAHA, 5-AZA (AZA), SAHA/AZA combination or untreated (CT). β-Actin was used as loading control. Histograms represent the mean plus SD of the densitometric analysis derived from three experiments and expressed as the ratio between cleaved Capase3 and β-Actin; *p*-value: * < 0.05; ** < 0.01; and **** < 0.0001, as calculated by ANOVA test.

Since it has been reported that histone deacetylation can be influenced by DNA methylation [14], here we investigated whether the DNA demethylating agent 5-AZA could influence SAHA-induced acetylation in pancreatic cancer cell lines carrying C176S (PaCa44) and R280K (PT45) mutp53. As shown in Figure 1A, the combination of SAHA/5-AZA induced a more intense pan-lysine acetylation compared to SAHA alone, in both cell lines, as evaluated by using a monoclonal antibody able to recognize acetylated lysine. This result was subsequently confirmed by evaluating histone H3 acetylation using a pan-acetyl anti-H3 antibody (Figure 1B). We then wondered whether this effect induced by SAHA/5-AZA could correlate with a higher cytotoxic effect compared to SAHA alone, given that the anti-tumor effect of HDACi involves an increase in acetylation. We found that the survival of both Paca44 and PT45 was reduced by SAHA and more strongly by the SAHA/5-AZA combination (Figure 1C). The enhanced cytotoxicity correlates with a stronger activation of caspase 3 in cells treated with SAHA/5-AZA, suggesting the occurrence of apoptotic cell death in cells subjected to this drug combination (Figure 1D).

### 2.2. SAHA/5-AZA Induces a Stronger Increase in MT2A Expression than SAHA

**Figure 2:**
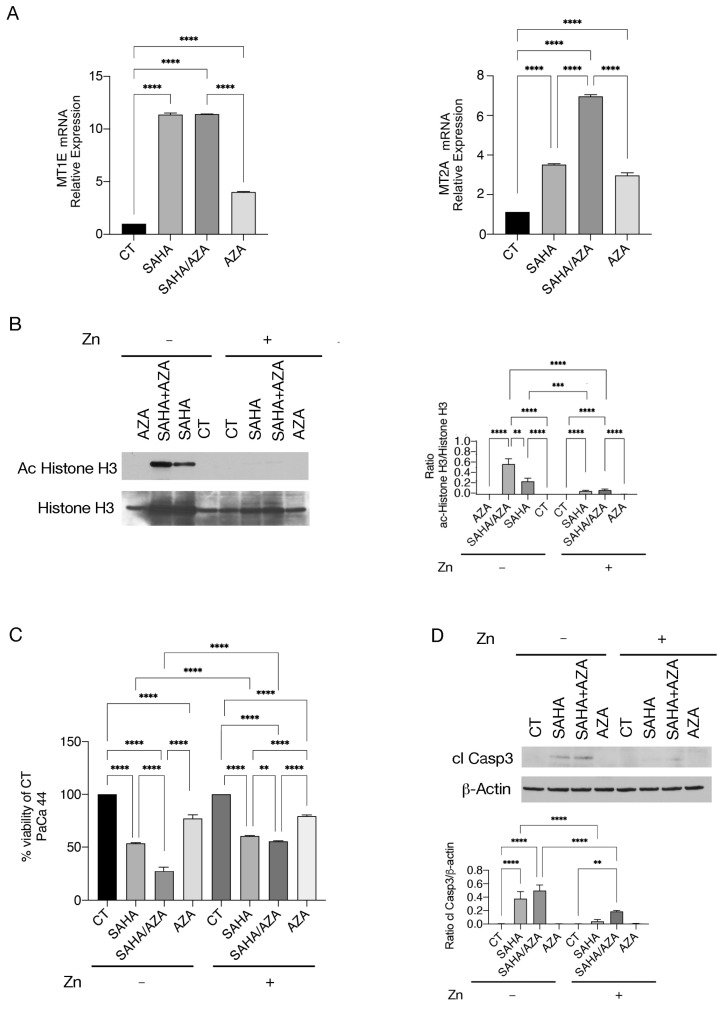
SAHA/5-AZA enhances MT2A expression more strongly than SAHA. (**A**) mRNA expression of MT1E and MT2A was evaluated by qRT-PCR by using the SYBR Green assay (Applied Biosystems, Carlsbad, CA, USA) with a StepOne instrument and 7500 Fast Real-Time PCR System (Applied Biosystems). The data are expressed relative to the reference gene (βActin). Histograms represent the mRNA expression levels of MT1E and MT2A. Data are represented as the mean relative to the control plus SD. **** *p* < 0.0001; (**B**) To evaluate the role of zinc on acetylation, ZnCl_2_ (50 mM) was added to PaCa44 cells treated with SAHA, 5-AZA (AZA), SAHA/AZA combination or untreated (CT) and acetylated Histone H3 was investigated by western blot analysis. Histograms represent the mean plus SD of the densitometric analysis of the ratio of Acetylate Histone H3/Histone H3; *p*-value: ** < 0.01; *** < 0.001 and **** < 0.0001, as calculated by ANOVA test. (**C**) cell survival was assessed by Trypan blue assay in PaCa44 cell line treated with SAHA, 5-AZA (AZA), SAHA/AZA combination or untreated (CT) in the presence or in the absence of ZnCl_2_. The histograms represent the percentage of cell viability relative to the control; data are shown as the mean plus SD of more than three experiments *p*-value: ** < 0.01 and **** < 0.0001, as calculated by ANOVA test. (**D**) Cleaved Caspase 3 (cl Casp3) was evaluated by Western Blot analysis in PaCa44 treated as described in panel B. Histograms represent the mean plus SD of the densitometric analysis derived from three experiments and expressed as the ratio between cleaved Capase3 and β-Actin; *p*-value: ** < 0.01; and **** < 0.0001, as calculated by ANOVA test.

To investigate the possible mechanisms leading to the increased acetylation induced by SAHA/5-AZA compared to SAHA, we investigated the expression of metallothioneins (MTs). A previous study has in fact demonstrated that DNA methyltransferase inhibitors and HDAC inhibitors can synergistically activate the promoter of MTs [15], proteins that can sequester the zinc ions necessary for the activity of zinc-dependent HDACs. To investigate changes in MT expression, we performed qRT-PCR on two of them, namely MT1E and MT2A. As shown in Figure 2A, MT1E was upregulated by both SAHA and SAHA/5-AZA, while MT2A was upregulated more strongly by the combined SAHA/5-AZA treatment. To evaluate whether zinc sequestration by MT could underlie the stronger acetylation mediated by SAHA/5-AZA, we added zinc chloride before starting treatment and found that histone H3 acetylation (Figure 2B), the cytotoxic effect (Figure 2C), and caspase 3 cleavage (Figure 2D) were reduced. These data suggest that zinc reduction could contribute to the greater acetylation and cytotoxicity mediated by SAHA/5-AZAcompared to SAHA.

### 2.3. SAHA/5-AZA Increases mutp53 Acetylation Further Promoting Its Proteasomal Degradation

**Figure 3:**
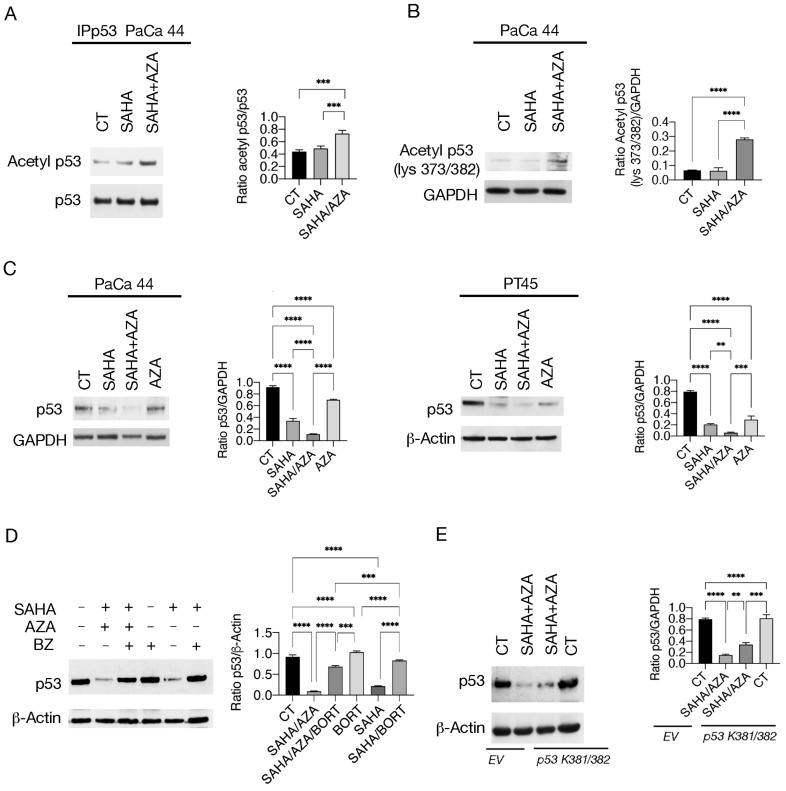
SAHA/5-AZA increases acetylation and proteasomal degradation of mutp53. (**A**,**B**) Acetylation of p53 as evaluated (**A**) by immunoprecipitation of p53 and blotting with an antibody against acetylated lysine; *p*-value: *** < 0.001 and (**B**) by western blot by using an anti-acetyl 373/382 p53 antibody in SAHA or SAHA/AZA-treated or untreated PaCa44 cells. GAPDH was used as the house-keeping control. Histograms represent the mean plus SD of the densitometric analysis derived from three experiments and expressed as the ratio between acetyl p53/p53 and acetyl p53 and GAPDH; *p*-value: **** < 0.0001, as calculated by ANOVA test. (**C**) p53 was evaluated by Western Blot analysis in SAHA or SAHA/AZA and AZA-treated or untreated (CT) PaCa44 and PT45 cells. GAPDH and β-Actin were used as loading controls. Histograms represent the mean plus SD of the densitometric analysis derived from three experiments and expressed as the ratio between p53 and loading controls; *p*-value: ** < 0.01; *** < 0.001; and **** < 0.0001, as calculated by ANOVA test. (**D**) proteasomal degradation of mutp53 by SAHA and SAHA/5-AZA as evaluated by Bortezomib (BZ) supplementation (10 nM), as reported in MM. β-Actin was used as the loading control. Histograms represent the mean plus SD of the densitometric analysis derived from three experiments and expressed as the ratio between p53 and β-Actin; *p*-value: *** < 0.001; and **** < 0.0001, as calculated by ANOVA test. (**E**) p53 expression as evaluated by western blot in PaCa44 cells, transfected with *p53 K381/382R* vector or with empty vector (EV) and treated with SAHA/AZA combination or left untreated. β-Actin was used as loading control. Histograms represent the mean plus SD of the densitometric analysis derived from three experiments and expressed as the ratio between p53 and β-Actin; *p*-value: ** < 0.01; *** < 0.001; and **** < 0.0001, as calculated by ANOVA test.

Similar to wtp53, mutp53 can be acetylated on multiple lysine residues, and this post-translational modification has been reported to promote the degradation of mutp53 (R175H), reducing cell survival in NSLC [10]. Here we studied the acetylation of mutp53 after treatment with SAHA and SAHA/5-AZA and, by immunoprecipitating mutp53 and blotting with anti-pan-lysine antibody, found that its acetylation increased (Figure 3A) This result was then extended by using a monoclonal antibody directed against p53 acetylated at 373/382 lysine (Figure 3B), according to knowledge that 382 residue can be acetylated by HDAC6 inhibitors [5] and to the fact that SAHA can inhibit HDAC6 among other HDACs [17]. We observed that acetylation of mutp53 correlated with stronger downregulation of this protein by SAHA/5-AZA compared to SAHA alone, in both PaCa44 and PT45 cells (Figure 3C). However, single treatment with SAHA, although to a lesser extent, reduced the expression level of mutp53 too. This effect could be correlated with increased acetylation of HSP90, as HDAC6 inhibition has been reported to acetylate it and reduce the ability of this chaperone to stabilize mutp53 [8]. As shown in Supplementary Figure S1, both SAHA and SAHA/5-AZA enhanced HSP90 acetylation and this effect may be involved in the reduction of mutp53 induced by SAHA. The downregulation of mutp53 by SAHA or SAHA/5-AZA was counteracted by Bortezomib (Figure 3D), which suggests that this protein underwent proteasomal degradation after these treatments. To investigate the contribution of mutp53 acetylation to the stronger mutp53 degradation observed in SAHA/5-AZA-treated cells, we transfected PaCa44 cells with *p53 K381/382R* vector. We found that the expression level of mutp53 was partially rescued following SAHA/5-AZA combination treatment (Figure 3E). Overall, these results suggest that SAHA/5-AZA-mediated acetylation of mutp53 contributed to its proteasomal degradation in pancreatic cancer cells. Next, we evaluated whether the transfection by *p53 K381/382R* vector could reduce the cytotoxic effect induced by SAHA/5-AZA, as mutp53 often acquires pro-survival properties [11]. We found that SAHA/5-AZA combination treatment was less effective in compromising tumor cell survival in cells transfected with *p53 K381/382R* vector compared to the empty control vector (Supplementary Figure S2). These results suggest that acetylation of mutp53 by SAHA/5-AZA contributed to its degradation, increasing the cytotoxic effect against pancreatic cancer cells.

### 2.4. SAHA/5-AZA Interrupts the Positive Crosstalk between c-Myc and p53

**Figure 4:**
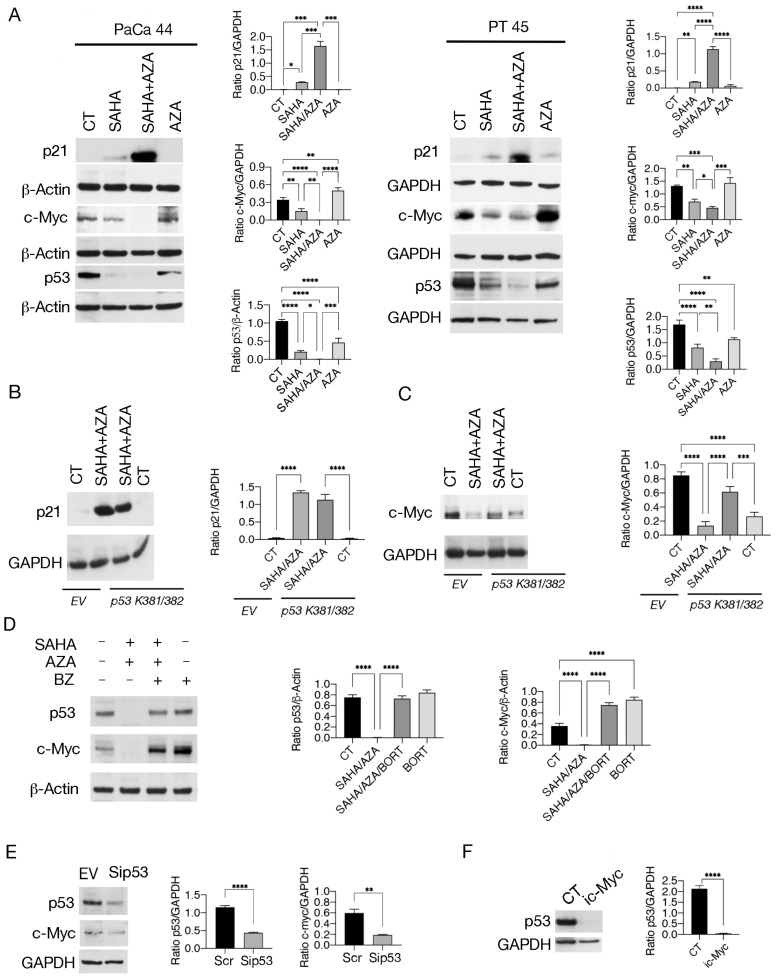
SAHA/5-AZA interferes with the positive crosstalk between c-myc and p53. (**A**) p21, c-Myc and p53 were evaluated by western blot analysis in PaCa44 and PT45 cells treated with SAHA, 5-AZA (AZA), SAHA/AZA combination or untreated (CT), as above reported. β-Actin and GAPDH were used as the loading controls. The histograms represent the mean plus SD of the densitometric analysis derived from three experiments and expressed as the ratio of p21/β-Actin, c-Myc/β-Actin and p53/β-Actin for PaCa44 cells and as the ratio of p21/GAPDH and c-Myc/GAPDH for PT45; *p*-value: * < 0.05; ** < 0.01; *** < 0.001; and **** < 0.0001, as calculated by ANOVA test. (**B**,**C**) PaCa44 cells, transfected with p53 K381/382R vector or with empty vector (EV) were treated with SAHA/AZA combination or untreated and p21 (**B**) and c-Myc (**C**) were evaluated by western blot. GAPDH was used as the loading control. The histograms represent the mean plus SD of the densitometric analysis derived from three experiments and expressed as the ratio between p21/GAPDH and c-Myc/GAPDH; *p*-value: *** < 0.001; and **** < 0.0001, as calculated by ANOVA test. (**D**) c-Myc expression level following Bortezomib (BZ) supplementation, added during the last 12 h of treatments. β-Actin was used as loading control. Histograms represent the mean plus SD of the densitometric analysis derived from three experiments and expressed as the ratio between p53/β-Actin and c-Myc/β-Actin; *p*-value: **** < 0.0001, as calculated by ANOVA test. (**E**) PT45 cells were p53-silenced (Sip53) or treated or with empty vector (EV) and p53, and c-Myc expression was evaluated by western blot analysis. GAPDH was used as the loading control. The histograms represent the mean plus SD of the densitometric analysis derived from three experiments and expressed as the ratio between p53/GAPDH and c-Myc/GAPDH; *p*-value: ** < 0.01 and *** < 0.001; as calculated by Student’s *t*-test. (**F**) p53 was evaluated in PT45 cells treated with c-Myc inhibitor (i c-Myc) (50 μM) for 48 h by western blot analysis. GAPDH was used as the loading control. The histograms represent the mean plus SD of the densitometric analysis derived from three experiments and expressed as the ratio between p53/GAPDH and c-Myc/GAPDH; *p*-value: **** < 0.0001; as calculated by Student’s *t*-test.

Subsequently, we observed that the SAHA/5-AZA combination more strongly upregulated p21 compared to SAHA, in correlation with the increased reduction of mutp53 (Figure 4A). As shown in Figure 4B, the transfection with *p53 K381/382R vector* partially counteracted this effect, suggesting that acetylation and subsequent degradation of mutp53 by SAHA/5-AZA treatment was contributing to the upregulation of p21. Interestingly, we then found that the expression of c-Myc was reduced by SAHA and further downregulated by the SAHA/5-AZA combination (Figure 4A). This effect may play a role in the cytotoxicity of this treatment, as c-Myc is known to be overexpressed in pancreatic cancer and its expression to correlate with tumor aggressiveness [18]. Downregulation of c-Myc occurred to a lesser extent in SAHA/5-AZA-treated tumor cells transfected with *p53 K381/382R vector* (Figure 4C), suggesting that also its reduction was dependent on mutp53 acetylation and degradation. As shown in Figure 4D, the proteasome inhibitor Bortezomib rescued the expression level of c-Myc, which suggests that this oncogenic protein was also degraded through the proteasomal pathway. We then assessed whether reduction of mutp53 expression might be sufficient to downregulate c-Myc and observed that its silencing did reduce the expression level of c-Myc, regardless of the effect of acetylation (Figure 4E). In agreement with these results, previous studies have reported that different p53 mutants sustain c-Myc expression [19,20]. Interestingly, the pharmacological inhibition of c-Myc in turn downregulated mutp53 in pancreatic cancer cells (Figure 4F), as previously reported [21], indicating that mutp53 and c-Myc establish a cross-talk that SAHA and the even better combination SAHA/5-AZA were able to interrupt.

### 2.5. SAHA/5-AZA Downregulates BRCA-1 and Induces DNA Damage in Pancreatic Cancer Cells

**Figure 5:**
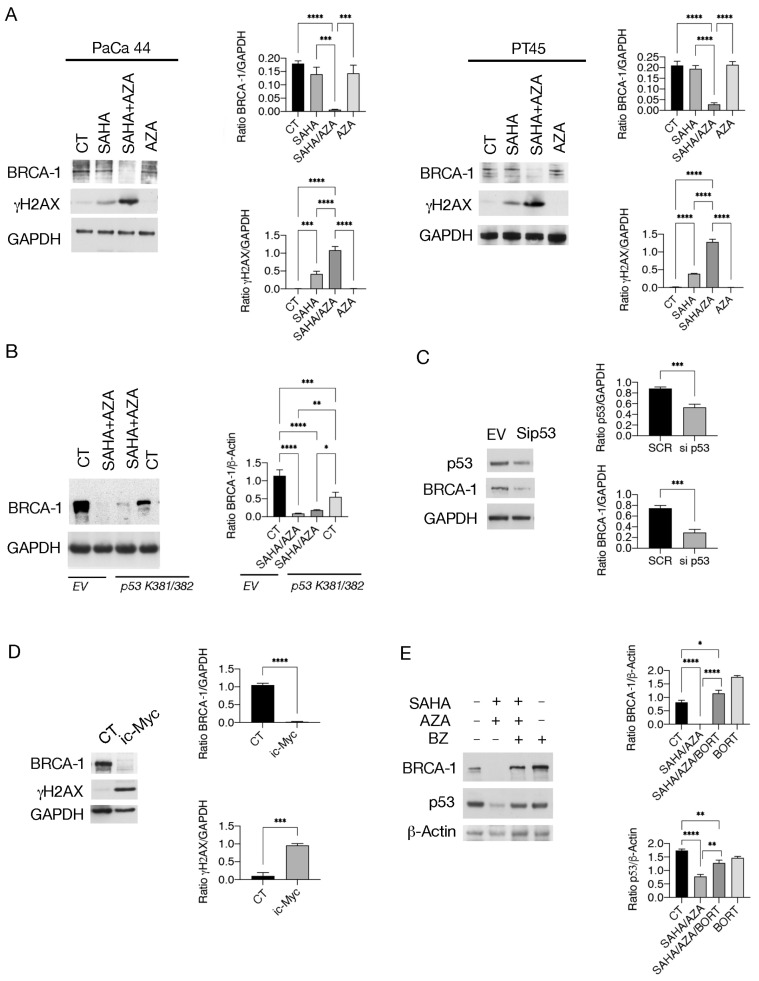
SAHA/5-AZA downregulates BRCA-1 and induces a strong DNA damage in pancreatic cancer cells. (**A**) BRCA1 and γH2AX were evaluated by western blot in PaCa44 and PT45 cells treated with SAHA, 5-AZA (AZA), SAHA/AZA combination or untreated (CT), as above reported. GAPDH was used as loading control. The histograms represent the mean plus SD of the densitometric analysis derived from three experiments and expressed as the ratio of BRCA1/GAPDH and γH2AX GAPDH; *p*-value: *** < 0.001; and **** < 0.0001, as calculated by ANOVA test. (**B**) PaCa44 cells, transfected with *p53 K381/382R vector* or with empty vector (EV) were treated with SAHA/AZA combination or untreated and BRCA1 was evaluated by western blot. β-Actin was used as the loading control. The histograms represent the mean plus SD of the densitometric analysis derived from three experiments and expressed as the ratio between BRCA1/β-Actin; *p*-value: * < 0.05; ** < 0.01; *** < 0.001; and **** < 0.0001, as calculated by ANOVA test (**C**) PaCa44 cells were p53-silenced (Sip53) or treated with empty vector (EV) and p53 and BRCA1 expression was evaluated by western blot analysis. GAPDH was used as the loading control. The histograms represent the mean plus SD of the densitometric analysis derived from three experiments and expressed as the ratio between p53/GAPDH and BRCA1/GAPDH; *p*-value: *** < 0.001; as calculated by Student’s *t*-test. (**D**) BRCA1 and γH2AX were evaluated in PT45 cells treated with c-Myc inhibitor (i c-Myc) (50 μM) for 48 h by western blot analysis. GAPDH was used as the loading control. The histograms represent the mean plus SD of the densitometric analysis derived from three experiments and expressed as the ratio between p53/GAPDH and c-Myc/GAPDH; *p*-value: *** < 0.001 and **** < 0.0001; as calculated by Student’s *t*-test. (**E**) Proteasomal degradation of BRCA1 evaluated by Bortezomib (BZ) supplementation, added for the last 12 h of treatments. β-Actin was used as the loading control. The histograms represent the mean plus SD of the densitometric analysis derived from three experiments and expressed as the ratio between p53/β-Actin and BRCA-1/β-Actin; *p*-value: * < 0.05; ** < 0.01; **** < 0.0001, as calculated by ANOVA test.

Since c-Myc has been shown to protect against DNA damage, mainly by supporting homologous repair (HR) [22], here we investigated the impact of SAHA/5-AZA treatment on BRCA-1, a protein playing a key role in this DNA repair pathway [23]. As shown in Figure 5A, SAHA/5-AZA induced stronger downregulation of BRCA-1 than SAHA and, accordingly, DNA damage increased in these cells, as indicated by phosphorylation of H2AX (γH2AX) (Figure 5A). Next, we found that either the transfection with *p53 K381/382R* vector before exposing cells to SAHA/5-AZA (Figure 5B), or the silencing of p53 (Figure 5C) or the treatment by c-Myc inhibitor (Figure 5D), less efficiently counteracted the reduction of BRCA-1, suggesting that the disruption of mutual support between mutp53 and c-Myc contributed to BRCA-1 downregulation and to DNA damage induction in pancreatic cancer cells. The expression of BRCA-1 was also prevented by proteasome inhibition by Bortezomib (Figure 5E) suggesting that this protein, as reported for mutp53 and c-Myc, underwent proteasomal degradation following treatment by SAHA/5-AZA.

## 3. Discussion

DNA methylation, together with histone modifications, represent key epigenetic pathways regulating chromatin structure and function. In particular, histone deacetylation and DNA methylation in promoter regions are associated with chromatin compaction and therefore inhibition of gene transcription. Consequently, HDAC and DNMT inhibitors, by increasing histone acetylation and reducing methylation, can synergistically reactivate genes that are silenced in tumor cells, such as tumor suppressors [24]. These mechanisms may contribute to the increase of cytotoxicity when these drugs are used in combination against tumor cells [16]. Interestingly, an interaction between these epigenetic pathways has also been described, as DNA methylation can influence acetylation of histone and non-histone proteins, and acetylation can in turn influence DNA methylation, so that concomitantly targeting epigenetic changes increases their anti-cancer effect [25]. For example, DNA methylation may represent a mechanism to initiate histone deacetylases [26] and the methyl-CpG binding protein MeCP2 (MeCP2), a protein that binds to chromosomes in a methylation-dependent manner, associates to histone deacetylases, leading to local deacetylation of histones and other proteins involved in the regulation of gene transcription [27]. Furthermore, DNA methyltransferase 1 (Dnmt1) has been reported to possess histone deacetylase activity [14]. Therefore, according to these studies reporting that DNA demethylation can increase acetylation, here we found that 5-AZA increases SAHA-induced pan-lysine acetylation in pancreatic cancer cells. Such combined treatment enhanced also the acetylation of mutp53, further promoting its proteasomal degradation compared to SAHA, which to a lesser extent reduced the expression level of mutp53 in correlation with the increased acetylation of HSP90 [8]. As a possible underlying mechanism leading to increased acetylation compared to SAHA, we found that SAHA/5-AZA treatment upregulated MT2A, differently from MT1E, which was upregulated by both SAHA and SAHA/5-AZA treatments [28]. MTs bind zinc and act as an intracellular reservoir of this ion, which is necessary for the deacetylating activity of HDACs such as those inhibited by SAHA. Therefore, upregulation of MT2A by SAHA/5-AZA could sequester more zinc, further impairing HDAC activity. This hypothesis was supported by experiments in which zinc chloride supplementation partially rescued mutp53 expression in SAHA/5-AZA-treated tumor cells. In line with these findings, a previous study reported that dual treatment with DNA methyltransferase and HDAC inhibitors can induce stronger activation of the metallothionein promoter [15]. Understanding these molecular mechanisms is of fundamental importance since the increase in acetylation induced by SAHA/5-AZA correlates with the induction of a stronger apoptotic effect. This suggests that the combination of DNA demethylating agents and HDACi could be a promising strategy for the treatment of pancreatic cancer, an aggressive cancer that, in most cases, carries p53 mutations. Moreover SAHA/5-AZA, in addition to the stronger downregulation of mutp53, further reduced the expression of c-myc, whose role in oncogenesis, also in the context of pancreatic cancer, has been widely demonstrated [21,29]. However, c-Myc and mutp53 were interconnected, since the silencing of mutp53 downregulated c-Myc, and on the other hand, the inhibition of c-myc reduced the expression of mutp53. SAHA/5-AZA interrupted the criminal alliance between these oncogenic proteins, leading to the proteasomal degradation of both and strongly downregulated BRCA-1. The latter is a key molecule involved in DNA HR and, as a consequence, SAHA/5-AZA strongly increased DNA damage in pancreatic cancer cells.

In conclusion, this study shows that the inhibition of histone acetylation and DNA methylation more effectively increased protein acetylation, including that of mutp53, leading to an increase in its degradation. This resulted in a greater cytotoxic effect against pancreatic cells, also related to stronger downregulation of c-myc and BRCA-1 and induction of DNA damage. Since p53 is mutated in approximately 75% of pancreatic tumors and as no standard therapies based on mutp53 are available, a better knowledge of the molecular mechanisms that regulate its degradation is of fundamental importance for developing new therapeutic strategies. As this study suggests, epigenetic manipulations could help move in this direction.

## 4. Materials and Methods

### 4.1. Cell Cultures and Treatments

The human pancreatic cancer-derived cell line PaCa44 was obtained from Dr. M. von Bülow (University of Mainz, Mainz, Germany), and PT45 was obtained from Dr. H. Kalthoff (University of Kiel, Germany). They were cultured in RPMI 1640 medium (Sigma-Aldrich, St. Louis, MO, USA, R1780), supplemented with 10% fetal bovine serum (FBS) (Sigma-Aldrich, St. Louis, MO, USA), L-glutamine (2 mM) (Aurogene, Rome, Italy), and streptomycin/penicillin (100 μg/mL) (Aurogene, Rome, Italy) (complete medium) at 37 °C in a 5% CO_2_ humidified incubator. Cells were seeded into 6-well plates at a density of 2 × 10^5^/well in 2 mL of complete medium and were treated, singly or in combinations, for 48 h with Vorinostat (SAHA) (5 μM) (MedChemExpress, New York, NJ, 08852, USA, cat. HY-10221/CS-0589) and 5-Azacitidine (5-AZA) (40 nM) (Selleckchem, Cologne, Germany, cat. S1782) added every 24 h. In some experiments, PaCa44 cells were untreated, or treated with c-Myc inhibitor (i c-Myc) (50 μM) (Merckmillipore, Darmstadt, Germany, cat. 475956-10-MG) for 48 h. To investigate protein degradation by proteasome, Bortezomib (Bort) (10 nM) (Sigma-Aldrich, Burlington, MA, USA, 5.04314) was added during the last 12 h to SAHA, AZA, and SAHA/AZA combination treated and untreated cells. To evaluate the zinc effect, ZnCl_2_ (50 μM) (Sigma-Aldrich, Burlington, MA, USA, 208086) was added to SAHA, AZA, SAHA/AZA combination and untreated cell cultures.

### 4.2. Cell Assay Viability

After SAHA and AZA treatments, a trypan blue (Sigma-Aldrich, Burlington, MA, USA, 72571) dye exclusion assay was performed to determine the number of viable cells. Unstained cells (live cells) were counted by light microscopy using a Neubauer hemocytometer. The experiments were performed in triplicate and repeated at least three times.

### 4.3. Western Blot Analysis

After treatments, the cells were harvested, centrifuged at 1200 rpm for 5 min at RT, and cell pellet lysed in RIPA buffer (150 mM NaCl, 1% NP-40, 50 mM Tris-HCl (pH 8), 0.5% deoxycholic acid, 0.1% SDS, protease, and phosphatase inhibitors), as previously reported [30]. The protein concentration was determined by using Quick Start Bovine Serum Albumin (BSA) assay (Bio-Rad, Hercules, CA, USA), and 10 μg of protein was denatured in loading buffer by heating for 10 min at 70 °C and subjected to electrophoresis on 4–12% NuPage Bis-Tris gels (Life Technologies, Carlsbad, CA, USA) according to the manufacturer’s instruction. The gels were transferred to nitrocellulose membranes (Bio-Rad, Hercules, CA, USA) for 50 min in tris-glycine buffer, and then stained with Ponceau S staining solution (SERVA Electrophoresis GmbH, Heidelberg, Germany, 33427.01) to verify protein transfer. The blots were subsequently washed three times with 1× PBS-0.1% Tween 20 solution containing 2% of BSA and incubated with specific HRP-conjugated secondary antibody. After three washes, the membranes were subjected to ECL (Advansta, San Jose, CA, USA, #12045-D20).

### 4.4. Antibodies

The following primary antibodies were used in western blots: mouse monoclonal anti-acetylated lysine (1:500) (Santa Cruz Biotechnology Inc., Dallas, TX, USA, cat n. sc-32268), rabbit polyclonal anti- acetylated Histone H3 (1:500) (Invitrogen, by ThermoFisher Scientific, Wallthm, MA, USA, PA5-114693), rabbit monoclonal anti-Histone H3 (1:4000) (Cell Signaling, Danvers, MA, USA, cat n. 4499T), mouse monoclonal anti-caspase3 (1:300) (Santa Cruz Biotechnology Inc., Dallas, TX, USA, cat n. sc-56053), rabbit polyclonal anti-Acetyl-p53 (Lys373; Lys382) (1:500) (Merckmillipore, Darmstadt, Germany, 06-758), mouse monoclonal p53 (1:500) (Santa Cruz Biotechnology Inc., Dallas, TX, USA, cat n. sc-126), rabbit polyclonal anti-c-myc (1:500) (Proteintech, Manchester UK, cat. n. 10828-AP), rabbit monoclonal anti-p21 (1:500) (Cell Signaling, Danvers, MA, USA, cat n. 2947T), rabbit polyclonal anti-BRCA-1 (1:2000) (Proteintech, Manchester UK, cat. n. 22362-1-AP) mouse monoclonal anti-γH2AX (1:100) (Santa Cruz Biotechnology Inc., Dallas, TX, USA, cat n. sc-517348), and rabbit polyclonal anti-HSP90 (1:5000) (Proteintech, Manchester UK, cat. n. 13171-AP). Mouse monoclonal anti-βActin (1:10,000) (Sigma-Aldrich, St. Louis, MO, USA cat n. A5441) and mouse monoclonal anti-GAPDH (1:10,000) (Santa Cruz Biotechnology Inc., Dallas, TX, USA, cat n. A5316) were used to detect the loading control. All primary and secondary antibodies were diluted in 1× PBS-0.1% Tween20 solution containing 2% of BSA (SERVA Electrophoresis GmbH, Heidelberg, Germany). The antibodies used in these experiments are listed in the following table.
**Antibody****Isotype****Species****Diluition Ratio****Company****Catalog**anti-P53 (DO-1)MonoclonalMouse(1:500) Santa Cruz Biotechnology sc-126anti-Ac-lysine (AKL5C1)MonoclonalMouse(1:500) Santa Cruz Biotechnology sc-32268anti-γH2AX (Ser 139)MonoclonalMouse(1:100) Santa Cruz Biotechnology sc-517348anti-Histone H3 ac PolyclonalRabbit(1:500) InvitrogenPA5-114693anti-Histone H3 (D1H2)PolyclonalRabbit(1:4000)Cell Signaling4499Tanti-c-myc PolyclonalRabbit(1:500) Proteintech10828-APanti-p21(12D1) PolyclonalRabbit(1:500) Cell Signaling2947Tanti-Acetyl-p53 (Lys373; Lys382) PolyclonalRabbit(1:500) Merckmillipore06-758anti-BRCA-1 PolyclonalRabbit(1:2000) Proteintech22362-1-APanti-βActin (AC-74)MonoclonalMouse(1:10,000) Sigma-AldrichA5441anti-GAPDH (G-9)MonoclonalMouse(1:10,000) Santa Cruz Biotechnology A5316anti-caspase3 (31A1067)MonoclonalMouse(1:300)Santa Cruz Biotechnology sc-56053

### 4.5. RNA Isolation and Quantitative Real-Time PCR Analysis

Total RNA was extracted from cells by using TRIzReagent (Invitrogen, Carlsbad, CA, USA) in accordance with manufacturer’s instructions. PCR analyses were carried out using the following specific oligonucleotide:

MT2A forw: 5′-CCGACTCTAGCCTCTT-3′
MT2A rev: 5′-GTGGAAGTCGCGTTCTTTACA-3′
MT1E forw:5′-GCTTGTTCGTCTCACTGGTG-3′
MT1E rev: 5′-CAGGTTGTGCAGGTTCTA-3′
ACT forw: 5′-TCACCCACACTGTGCCATCTACGA-3′
ACT rev: 5′-CAGCGGAACCGCTCATTGCCAATGG-3′.

Transcripts were measured by real-time PCR using the SYBR Green assay (Applied Biosystems, Carlsbad, CA, USA) with a StepOne instrument and 7500 Fast Real-Time PCR System (Applied Biosystems). All primer sets worked under identical quantitative PCR cycling conditions with similar efficiencies to obtain simultaneous amplification in the same run. PCR protocol was set at 95 °C—2 min, the steps were repeated for 40 times: (95 °C—10 s; 55 °C—20 s; 72 °C—1 s) and then 4 °C-infinite The 2^−ΔΔC*T*^ method for relative quantitation of gene expression was used to determine mRNA expression levels. *β-actin* gene expression was used as endogenous controls to compare mRNA expression. All reactions were run in triplicate.

### 4.6. Knockdown of p53 and Transfection with p53 K381/382R Vector

P53 silencing was performed by transfection with p-Super empty vector (EV) or with pSUPER-p53 vector (Sip53), according to [31]. pcDNA3-wtp53, K382R (lysines 381 and 382 mutated to arginine) vector or empty vector were prepared according to what was previously reported [32] and transfected in Paca44 cell line. In both cases, cells were transfected using Lipofectamine RNAiMAX Reagent (Thermo Fisher Scientific, USA, 13778075). Briefly, PaCa44 cand PT45 cells were seeded into 6-well plates at a density of 2 × 10^5^ cells per well and, the following day, transfected with the above indicated vectors or empty-GFP vector, as control. Cells were collected after 72 h of transfection for subsequent analysis.

### 4.7. Immunoprecipitation Assay

To perform the immunoprecipitation assay, 1.5 × 10^6^ PaCa44 cells were treated, singly or in combinations, for 48 h with Vorinostat (SAHA) (5 μM) (MedChemExpress, NJ 08852, USA) and 5-Azacitidine (5-AZA) (40 nM) (Selleckchem, Cologne, Germany) added every 24 h. After treatments, cells were collected, lysed in 500 μL of RIPA and centrifuged at 14,000 rpm for 30 min at 4 °C. Pre-clearing lysate was performed by adding 20 μL of G plus agarose (Santa Cruz Biotechnology Inc., Dallas, TX, USA cat. n. sc-35597) for 1 h at 4 °C. For protein immunoprecipitation, 5 μL of anti-p53 or anti-Ac-Lysine antibodies (Santa Cruz Biotechnology Inc., Dallas, TX, USA cat. n. sc-126 and sc-32268) were added to the cellular extract, and samples were incubated overnight at 4 °C in a constant rotation movement. The day after 20 μL of protein G plus agarose was added to each sample for 1.5 h at 4 °C in a constant rotation. Precipitated proteins were collected by centrifugation, washed three times in lysis buffer, and analyzed by western blot analysis.

### 4.8. Densitometric Analysis

The quantification of protein bands was performed by densitometric analysis using the Image J software (1.47 version, NIH, Bethesda, MD, USA), which was downloaded from the NIH website (http://imagej.nih.gov, accessed on 10 February 2022).

### 4.9. Statistical Analysis

Results are represented by the mean plus standard deviation (S.D.) of at least three independent experiments, and statistical analyses were performed with Graphpad Prism^®^ software (version 9; Graphpad Software Inc., La Jolla, CA, USA). Student’s *t*-test or a nonparametric one-way ANOVA test was used to demonstrate statistical significance. The difference was considered statistically significant when the *p*-value was: * < 0.05; ** < 0.01; *** < 0.001; and **** < 0.0001.

## Data Availability

The datasets generated and analyzed during the current study are available from the corresponding author upon reasonable request.

## Supplementary Materials

The following supporting information can be downloaded at: https://www.mdpi.com/article/10.3390/ijms25137020/s1.
