# Supplementary material for: SAHA/5-AZA Enhances Acetylation and Degradation of mutp53, Upregulates p21 and Downregulates c-Myc and BRCA-1 in Pancreatic Cancer Cells
Source: Int J Mol Sci. 2024 Jun 27;25(13):7020. doi: 10.3390/ijms25137020 (PMC11241381; doi:10.3390/ijms25137020)
Supplement: Supplementary file 1 [file ijms-25-07020-s001.zip › ijms-3054067-supplementary.pdf]

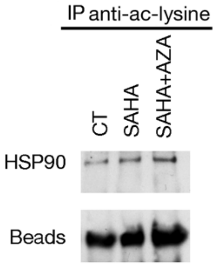

Supplementary Figure S1

Supplementary Figure S1 HSP90 acetylation evaluated after immunoprecipitation with anti-pan-lysine acetylated and blotted with anti-HSP90 antibody by western blot analysis in SAHA or SAHA/AZA-treated or untreated (CT) PaCa44 cells. Beads were used as loading control.

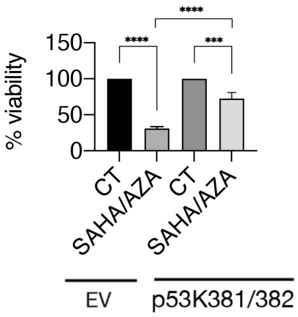

Supplementary Figure S2

Supplementary Figure S2 Cell survival was assessed by Trypan blue assay in PaCa44 cells, transfected with p53 K381/382R vector or with empty vector (EV) and treated with SAHA/AZA combination or untreated (CT). The histograms represent the percentage of cell viability relative to the control; data are shown as the mean plus SD of more than three experiments p-value: *** <0.001; and **** <0.0001, as calculated by Student's *t*-test.
